# Redefining social support: a scoping review of the effects of digital technologies on the social support of older workers

**DOI:** 10.1186/s12889-025-26155-w

**Published:** 2026-01-14

**Authors:** Cristina Maria Tofan, Anna Ševčíková, Nilufer Korkmaz Yaylagul, Gunilla Kulla, Günay Yıldızer, Murat Anil Mercan, Hande Barlın, Yang Gu, Kerstin Nilsson, Diana Alecsandra Grad, João Rocha Gomes, Jeroen Spijker

**Affiliations:** 1 Psychology and Educational Sciences Department, Gheorghe Zane Institute for Economic and Social Research, Romanian Academy, Iasi Branch, Iasi, Romania; 2 Department of Sociology, Social Work and Human Resources, Faculty of Philosophy and Social-Political Sciences, Alexandru Ioan Cuza University of Iasi, Iasi, Romania; 3 Psychology Research Institute, Faculty of Social Studies, Masaryk University, Brno, Czechia; 4 Gerontology Department, Faculty of Health Sciences, and in Ageing Research Center, Akdeniz University, Antalya, Türkiye; 5 Department of Health and Caring Sciences, Faculty of Health and Social Sciences, Western Norway University of Applied Sciences, Forde, Norway; 6 Eskişehir Technical University, Faculty of Sport Sciences, Eskişehir, Türkiye; 7 Department of Economics, Faculty of Economics and Administrative Sciences, Yildiz Technical University, Istanbul, Türkiye; 8 Department of Economics, Faculty of Business Administration, Gebze Technical University, Gebze, Türkiye; 9 Work, Employment, Management and Organisation Department, Business School, University of Leicester, Leicester, United Kingdom; 10 Division of Occupational and Environmental Medicine, Lund University, Lund, Sweden; 11 Department of Public Health, Kristianstad University, Kristianstad, Sweden; 12 Department of Public Health, Babes-Bolyai University, Cluj-Napoca, Romania; 13 Department of Community Medicine, Health Information and Decision, Faculty of Medicine, University of Porto, Porto, Portugal; 14 Universitat Internacional de Catalunya, Barcelona, Spain, and Centre d’Estudis Demogràfics, Bellaterra, Spain

**Keywords:** Digitalisation, Digital technologies, Social support, Older workers, Health

## Abstract

**Introduction:**

The rapid digitalisation of workplaces presents both challenges and opportunities for older workers. This scoping review examines how digital technologies impact social support for older workers, focusing on emotional, informational, and instrumental support within professional environments. While social support is critical for well-being and productivity in ageing workforces, the effects of digitalisation on social support dynamics remain insufficiently understood.

**Methods:**

Following Joanna Briggs Institute and PRISMA-ScR guidelines, a comprehensive search strategy was conducted across databases like ERIH, Web of Science, Scopus, and PubMed from anytime to 2023 to identify peer-reviewed studies involving digital technologies used by older workers, generally considered as workers aged 50 years or older. Covidence software facilitated the screening of over 5000 scientific papers, study selection, and data extraction, and the Mixed Methods Appraisal Tool (MMAT) assessed quality. Findings were synthesized through descriptive statistics and narrative analysis.

**Results:**

Forty-three studies met inclusion criteria. Digital technologies were found to enhance various forms of social support: remote work tools, messaging apps, and telemedicine platforms facilitated emotional connection and informational exchange. However, digitalisation also introduced barriers, some older workers reported isolation, reduced informal contact, and technostress, underscoring disparities in digital literacy and adaptation.

**Discussion:**

Digitalisation exerts a dual impact on social support for older workers: it can strengthen professional connectedness yet also heighten vulnerability to stress and exclusion. Targeted digital literacy initiatives and sustained managerial engagement are crucial to ensure that technology enhances, rather than undermines, well-being and productivity among ageing employees.

**Supplementary Information:**

The online version contains supplementary material available at 10.1186/s12889-025-26155-w.

## Background

The world is witnessing multiple transitions. Populations and workforces are ageing rapidly, particularly across the Organization for Economic Cooperation and Development (OECD) countries [1], while the accelerated adoption of digital technologies is transforming working life [2]. National governments and European and international organizations such as the European Union (EU), OECD, the World Health Organization (WHO), and the United Nations (UN), are increasingly promoting extended working life, not only for the sake of social security systems, but especially for healthy and active ageing [1, 3–5]. These developments highlight the importance of sustainable working lives, in which social support may be essential for retaining older employees and safeguarding their health and well-being amid a rapidly digitalising labour market [6, 7].

In this study, older workers are defined as people over the age of 50 [8] due to age-related declines in physical functioning and longer recovery times [9]. Furthermore, people over 50 are likely to experience ageism at work, especially when trying to re-enter the labour market [6, 10]. Even so, we are aware that age cut-off points are a multi-perspective issue influenced by psychological perception, social norms, and economical reasoning [10] argue that there is no universal accepted age that defines an “older worker”. Reported cut-off points vary greatly depending on the type of occupation ranging between 28 and 75 years old, with an average of 52.4 years.

Social support is commonly conceptualized in two forms: implicit and explicit. Implicit support refers to the reassurance derived from the mere presence or awareness of close others, without disclosing personal problems, whereas explicit support involves actively engaging one’s social network to share and discuss stressful experiences [7] Regardless of this distinction, previous research consistently identifies social support as a critical factor in sustaining a healthy working life across all age groups [11]. For example, a recent scoping review analysing job demands and resources mediated by digital platforms highlight social support as very important at the group or organizational level due to its interactional functions [12]. The social support is part of the resources and refers to the degree to which individuals feel valued by colleagues, supervisors, and the organization in which they work. However, as technological advancements reshape society, these changes also redefine the environment of work, opening new avenues for supporting an ageing workforce. In the context of accelerating workplace digitalisation and an ageing labour force, it is important to examine how digital technologies (e.g., web-based platforms, smartphone, computers) affect older workers access to social support, a key-determinant of well-being, adaptation and continued participation in the workforce.

The concept of social support itself is not straightforwardly defined, it is often used as an umbrella term referring to how relationships foster well-being, self-esteem, and other health indicators [13]. For example, Cobb’s [14] view is that social support provides information on how someone is cared for and acts as a defence mechanism against the impact of stress on health. He argues that social support is an important ally for health and reduces the time needed to manage stress. Lakey and Cohen [15] introduced three influential theoretical perspectives on social support: the stress and coping approach, the social constructionist approach, and the relationship approach. The stress and coping perspective suggest that social support enhances well-being by buffering individuals against the detrimental impacts of stress. In contrast, the social constructionist viewpoint argues that support enhances well-being by fostering self-esteem and self-regulation, irrespective of the presence of stress. The relational perspective proposes that the health outcomes of social support cannot be disentangled from the relational dynamics that often accompany support, including companionship, intimacy, and low social conflict. Finally, these perspectives highlight different mechanisms that can explain the connection between social support and health, and a potential role of digital tool in this association.

According to LaMeres [16], digital technologies are used for data manipulation, storage, transmission, and processing in digital format, all aimed at enhancing quality of life (e.g., web-based platforms, smartphones, computers, digital cameras, digital videos/audios, etc.). These technologies are increasingly recognised as offering a new form of social support, although no clear definitions exist for this type due to the diversity of technologies involved. In this respect, digital technologies have been examined as vehicles for providing new form of social support. (i.e., online social support), and evidence suggests that they influence health outcomes for older adults in similar ways to traditional forms of support [17] through companionship, coordination, maintaining ties, and casual conversations. Understanding the role of online social support in a digitalized labour market is important as digital technologies also present challenges for older workers. Both negative and positive effects are observed for older workers, and research does not always differentiate between effects for older adults and older workers. For example, Nimrod [18] identifies technostress resulting from interactions with information and communication technology (ICT), as a threat to older adult’s well-being. Similarly, Alcover et al. [19] argue that digitalisation (i.e., Artificial Intelligence (AI), robotics, automation) can increase job insecurity or negatively affects older workers’ wellbeing, as they often lack ICT skills or need more time to solve tasks involving ICT compared to younger workers. Finally, other research results indicate that social support helps older adults learn to use digital technologies (i.e., tablet computers;.

Nick et al. [20] propose measuring online social support, by categorising it according to its functional roles and purposes. They identify four main types: esteem/emotional support, social companionship, informational support, and instrumental support. Esteem/emotional support involves conveying acceptance, intimacy, care, liking, respect, and similar emotions through verbal and nonverbal cues. Social companionship support fosters a sense of belonging through actions that express inclusiveness or involve spending time together. Informational support encompasses sharing advice, feedback, knowledge, and resources. Instrumental support refers to the provision of practical assistance, including financial assistance, material help, task assistance, and taking on responsibilities. These categories provide a useful conceptual framework for understanding how digital technologies may facilitate different forms of social support in the workplace, particularly for older workers navigating digitalised work environments. Current theoretical frameworks of online social support build on previous influential perspectives, as many digital technologies now provide social support. For example, AI-based applications (apps) can assist older adults in their daily lives, for instance, by tracking and monitoring health indicators and cognitive functioning [21].

### Empirical research regarding social support in digitalized working settings

Early research on social support in online environments reveals mixed effects [22]. Findings suggest that social support via email and online chat is critical for health indicators, but social conditions influence media choice, and individuals continually assess the appropriateness of the social context. Francis et al. [23] uncovered in their qualitative analysis that coping with technical issues from regular ICT use also provides opportunities for both online and traditional social support.

Mendel et al. [24], through qualitative analysis, also highlight the mixed effects of the interaction between social support and digital technologies. While online social support related to fraud and phishing information may increase safety risks for older adults, mobile tools can also be used to raise awareness, encourage proactive behaviour, and foster learning to manage mobile safety challenges. Similarly, Marston and Musselwhite [25] advocate for the generally positive effects of technological tools in improving older people`s lives, while also identifying social barriers associated with learning to use such tools. Utz and Breuer [26] tested social network sites for providing social support and enhancing well-being. They found that those using social networks reported more online social support than those not using them, with users seeking more advice online. Likewise, Thompson and Atkins’s [27] found that technological tools facilitate the sharing of ideas, the creation of meaningful relationships, and the instantaneous sharing of information.

With respect to an ageing workforce, Thompson and Mayhorn [28] argue that digital technologies can serve as online support mechanisms for older workers by addressing physical demands, mobility concerns, visual acuity, workplace safety, memory limitations, new networking opportunities, and reducing age-related cues that prompt discrimination. Digital technologies are humanised, meaning they can become sources of social support or, conversely, a lack thereof (e.g. older workers may feel that computers restrict them), or alternatively, rely on them for assistance at work). For instance, researchers describe technological tools assisting older workers maintaining productivity, compensating for motor strength through using computer-aided manufacturing or using robotics to alleviate cognitive and physical stress by assisting with precise steps. Thus, the belief in digital social support could be seen as a type of social support shaped by digital technologies.

Moreover, social support is reinforced by social identity theory [29], and empirical research [30] indicates that both online and offline social support depend on group membership, which is particularly relevant for groups in the workplace who rely on mutual support, such as informal caregivers, as they benefit from shared experiences, resources, and a sense of belonging within their peer groups.

Previous research has often focused on the potential risks and vulnerabilities that digitalisation poses to ageing workers [18] or explored its positive impact on similar concepts to social support like social capital [31]. However, there has been little effort to identify types of social support provided through digital technologies specifically for older workers. This scoping review therefore aims to address this gap by exploring how digital technologies used in the work environment affect social support for older workers and identify the types of online social support that arise. Through a comprehensive review of existing research, this scoping review seeks to understand the role of digital technology in enhancing social support for older workers by addressing the following questions: Which digital technologies are most used by older workers and their colleagues to stay connected? How are these digital technologies being used by older workers? Do these digital technologies facilitate and mediate online social support? And how is the use of digital technologies for accessing social support is linked to the health of older workers? By synthesizing these findings, this study seeks to provide a comprehensive analysis of how digitalisation can be used to support and enhance the well-being of older workers.

## Methodology

This scoping review follows standard and recognised methodology for systematic reviews reporting by the Joanna Briggs Institute (JBI) [32] along with recommendations from Scoping Reviews checklists and the PRISMA extension for scoping reviews (PRISMA-ScR) flow diagram for new systematic reviews, which includes searches of databases and registers only [33]. We used Covidence software as a tool for managing references to facilitate title and abstract screening, to conduct full-text reviews and to support data extraction.

### Search strategy and definitions of key-concepts

A three-phase approach was implemented for the search strategy. First, we identified and defined the main concepts of digital technologies, online social support, and older workers. We followed the definitions of digital technologies provided by LaMeres [16]. Online social support refers to the use of digital technologies such as social media, online forums (i.e. professional groups/communities), and messaging platforms to manage difficulties, challenges, or serious problems. We considered three types of online social support: emotional support (key terms: empathy, encouragement, validation, concern, affection); informational support (key terms: advice, guidance); instrumental support (key terms: assistance with resources, financial assistance, online help, technical and organisational support). Additionally, we also paid attention to the availability of implicit social support, such as perception of workers of getting along with fellow workers. Social support through digital technology can take diverse forms, such as social media, online platforms, virtual communities, telemedicine platforms, online helpline services (e.g. psychological online services, professional IT or administrative online services, health apps, monitorization apps), facilitating easier connections and communication across distances. Older workers are defined as individuals aged 50 years and above who are employed. In this study, older workers are defined as those aged 50 years and above [8], reflecting evidence of age-related physical decline and slower recovery [9]. Beyond biological factors, this age group often encounters structural barriers such as workplace ageism, particularly during labour market re-entry [6, 10]. Yet, the notion of an “older worker” remains analytically fluid, shaped by psychological perceptions, social norms, and economic contexts [10].

Secondly, following the JBI methodology for scoping reviews the PCC (Participants, Concepts, Context) Framework was established. Accordingly, the participants (P) comprised studies involving workers aged 50 and above. The concept (C) includes studies on the use of digital technologies in the workplace that lead to social support. The Context (C) encompasses studies conducted in all workplace setting. The PCC framework informed our inclusion criteria. The search was limited to studies published in English and peer-reviewed scientific articles using quantitative, qualitative, or mixed methods, with no time constraints. Exclusion criteria were defined as following: as studies not concerning working individuals or not associated with work, studies using analogue technology (i.e., non-digital media such as analogue phone, or analogue fax machine), non-English articles, theoretical papers, books, book chapters, reviews, systematic reviews, reports, protocols, and non-peer-reviewed studies (Table 1).

**Table 1:** Inclusion and exclusion criteria

PCC Framework	Inclusion	Exclusion
Participants	Studies involving workers aged 50 and above	Non-working individuals or involving only individuals younger than 50
Concept	Studies on the use of digital technologies in the workplace leading to social support	Studies on analogue technology
Context	Studies conducted in all workplace contexts and in all countries English language articles Quantitative, qualitative, and mixed methods. Peer-reviewed scientific articles No time constraint regarding publication year	Studies not associated with employment and social support Non-English articles Other study types (theoretical papers, books, book chapters, reviews, systematic reviews, reports, protocols) Non-peer reviewed studies

Finally, search terms were identified based on an initial literature review regarding our main concepts, namely digital technologies, online social support, and older workers. A single syntax search was designed for all databases. Terms were identified as “social support” OR ”social assistance” OR ”emotional support” OR ”social aid” OR ”social advice” OR ”social guidance” OR ”instrumental support” OR ”information support” OR ”social help” OR ”financial assistance” OR ”online*” OR ”technical support” OR ”organisational support” OR ”affect* support”) AND (”digital*” OR ”platform*” OR ”apps*” OR ”tech*” OR ”social media” OR ”chat” OR ”online*” OR ”telemedicine” OR ”cyber” OR ”virtual” OR ”computerized” OR ”computerised” OR ”electronic” OR ”ICT”) AND (”old* work*” OR ”old* employee*” OR ”old* profession*” OR ”elder work*” OR ”aging work*” OR ”ageing work*” OR ”old* workforce” OR ”aged work*” OR ”senior work*”. Searches were undertaken in the databases ERIH, Web of Science, Scopus, PubMed, PsycINFO, Proquest in 04 May 2023 by three reviewers CT, MAM, DG. The search results were uploaded into Covidence.

### Study selection and data extraction

All studies identified through database searching were retrieved and then imported and stored in Covidence. Duplicates were automatically removed using Covidence’s built-in feature. The titles and abstracts of the identified studies were double-screened by 12 reviewers to determine if they met the inclusion criteria. The full texts of eligible studies were then retrieved and independently assessed by two reviewersin relation to our main research questions and the inclusion and exclusion criteria. Any disagreements were resolved through discussion or, when necessary, with theinvolvement of a third reviewer.We calibrated this process in Covidence through random allocation of studies, alongside initial calibration exercises and periodic agreement checks.

In the data extraction phase, 11 reviewers were involved. Two independent reviewers extracted the relevant information from the selected studies into the data extraction chart that we created in Covidence. Any disagreements between reviewers were resolved through discussion and validated by JS and CMT. The data chart included the following items: bibliographical reference, study location, data collection, information about invited and actual participants in the study, age used in the analysis or results of the study, information about whether the participants included both young and older people, and both older adults and workers, gender, population, social support, type of social support, the digital technologies used and type of digital technologies. Additionally, we extracted data about main theories and instruments used in the studies. Extracted data were exported to Microsoft Excel, analysed and presented using descriptive statistics, with a narrative summary presented below.

### Quality assessment

All the included studies were critically appraised for their methodological quality using the Mixed Methods Appraisal Tool (MMAT) Version 2018, developed by Hong et al. [34]. The MMAT provides comprehensive guidelines for assessing quality across five categories of study designs, including qualitative studies, randomized controlled trials, nonrandomized studies, quantitative descriptive studies, and mixed methods studies). The tool consists of two parts: a checklist with two initial screening questions to confirm the paper is an empirical study (i.e., the clarity of research questions and feasibility of a study to answer them), followed by five criteria for each study design category. The second part provides detailed explanations for each criterion to guide the assessment process.

Two reviewers independently appraised each study to minimize bias and ensure reliability. Discrepancies between reviewers were resolved through discussion to reach a consensus. For each study, the reviewers completed assessments that included two initial screening questions (“Yes” = 1 or “No” = 0) and then assessed a set of five criteria specific (“Yes”=1 or “No”=0) to the study design, as outlined by the MMAT. These criteria assess the appropriateness of the methodology, the adequacy of data collection methods, the relevance of the measurements to the research questions, and the coherence between data sources and analysis methods. Finally, for this review, we calculated the percentage of “Yes” responses for each study to provide an overview of the methodological quality. No studies were excluded based on this assessment. This quality percentage score was calculated by dividing the number of “Yes” answers by the number of applicable criteria and multiplying the result by 100. These quality percentage scores reflect the proportion of applicable MMAT criteria that each study met, indicating the level of methodological rigor according to the tool’s framework. Next, the quality percentage scores were averaged for the two initial screening questions and then for each study design to obtain mean quality scores. Studies with scores below 50% were classified as low quality, those between 50% and 79% as moderate quality, and those at or above 80% as high quality. This quantitative measure of quality will help discuss the reliability and validity of the findings from these studies within the broader context of our review.

## Results

### Selected studies

**Fig. 1:**
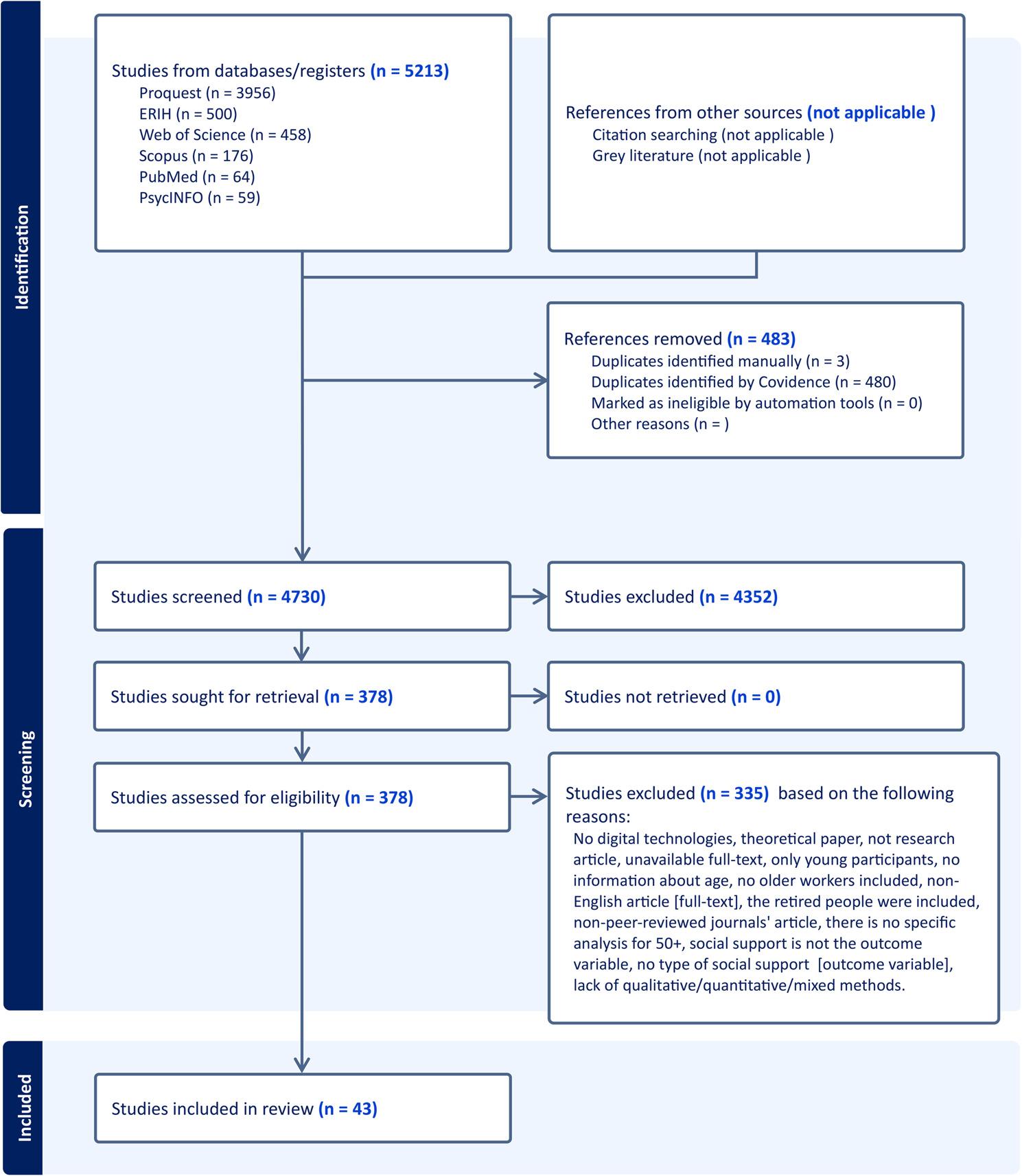
PRISMA flowchart for digitalization and social support for older workers

The initial database search identified 5,213 studies. After removing duplicates, 4,730 were screened for eligibility, with 378 retrieved for full-text review. Of these, 43 met our pre-established inclusion and exclusion criteria and were selected for data extraction. Studies excluded during the process are detailed in the PRISMA flow-chart (Fig. 1).

### Qualitative assessment

Overall, 81% of studies clearly stated research questions and collected appropriate data to answer them. The sample included 13 quantitative non-randomized studies of moderate quality (mean score: 66%), 9 quantitative descriptive studies of moderate quality (mean score: 50%), 2 randomized controlled trials bordering low to moderate quality (mean score: 40%), 9 qualitative studies of moderate quality (mean score: 78%), and 10 high quality mixed-method studies (mean quality: 80%). The lower quality of the quantitative descriptive studies was partly due to their frequent failure to report nonresponses or address the higher risk of nonresponse (*see Supplemental material_Quality assessment*).

### Characteristics of included studies

#### Authors and publishing and collection of data years

Of the 43 selected studies, the earliest two were published in 1998 [35] and in 2000 [36]. All others were published between 2016 and 2023. Of these studies, three used data from the year 2000 or earlier, none had data collected between 2001 and 2014, 12 were based on data collected between 2015 and 2019, and 21 studies used data collected in 2020 or later (in three of these studies, data were collected twice, once in each of the latter two periods). In 11 studies, the data collection year was not stated (see Table 2).

**Table 2:** Selection and description of studies (*N* = 43) for the association between digital technologies and social support specific to older workers

Author(s)	Year of data collection	Data collection method	*N* (invited/participants/follow-ups	Age used in the analysis/results*	both young & older workers	both older adults & workers	Gender**	Population	Social support (SS)	Type of SS	Digital technologies (DT)	Type of DT
Aborg et al. (1998) [35]	1991, 1992	Online survey and in-depth interviews	153/22/17	AR: 19–63	Yes	Yes	F	public institution workers	SS from colleagues and supervisors	Explicit	Data entry type of work	Explicit
Carayon and Karsh (2000) [36]	1994/1991-92.	Survey, semi-structured interviews	Agency A 149/47/NA Agency B 191/122/NA	MAge = 42.1; SD = 10	Yes	Yes	T	Workers in public institution	SS from colleagues and supervisors	Explicit	Image and non-image users	Implicit
Meyers and Bagnall (2016)	NS	Semi-structured interview	NS/10/NA	AR: 45–55	No	No	F, M	Older workers	Cognitive support	Implicit	Online learning (e.g., the use of technology, hypermedia, independent learning)	Explicit
Mohadis et al. (2016) [37]	NS	Interview	NS/10/NA	AR: 50–64	No	No	F, M	Workers	Social comparison and competition persuasive principles as SS	Implicit	FitSenior application	Explicit
Verbrugghe et al. (2016) [38]	2015	Survey	22,084/790/NA	AC: Up to 54, 55+	Yes	No	NS	Workers in the private sector	SS for sustainable employability	Implicit	Development of Healthy Workplaces for all Ages e-guide	Implicit
Arvola (2017) [39]	2016	Survey	NS/107/NA	AC: under 50, 50+	Yes	No	T	Workers	Getting on with fellow-workers and social networks	implicit	Teleworking and the extent that ICT devices and applications were used for work (PC, laptop, tablet PC, smart phone, MS Outlook, MS Office, social networks).	Explicit
Hauk et al. (2019) [40]	NS	Online survey	NS/1216/T2 = 840/T3 = 631	AR: 17–75	Yes	Yes	NS	Workers	Instrumental SS	Explicit	ICT tools	Explicit
Calderón-Gómez et al. (2020) [41]	2016	Survey	3000/2800/NA	AC: 16–34, 35–54, 55–64	Yes	Yes	F, M	internet users	Online communication with colleagues	Implicit	Online tools linked to the mobile phone and/or computer, including messaging services, social media, video conference apps, SMS, and email.	Explicit
Chandra et al. (2020) [42]	NS	Online survey	700/163/NA	MAge = 37.64, SD = 6.76.	Yes	No	F, M	service sector workers	Technological spatial intrusion and usefulness of ICT for workers	Explicit	ICT use	Implicit
De Leeuw et al. (2020) [43]	2017	Semi-structured interview	NS/10/NA	AR: 52–63	No	No	F, M	Workers	Health information	implicit	Health information technology; electronic health records and eHealth devices	Explicit
Handley and Outer (2020)	NS	Interview	NS/24/NA	AR: 48–58; MAge = 52.5	No	Yes	T	Knowledge workers	Lack of mentoring and acknowledgment at work through organisational decisions	Implicit	Workers from the IT software sector, film industry, and technology entrepreneurs.	Implicit
Middleton et al. (2020) [44]	2019–2020	Text messages	464/291/NA	AC: 17–30, 31–40, 41–50, 51–60, 61–70	Yes	No	F, M, T	Workers	Informational SS	Implicit	@Work (app intervention through text messages)	Explicit
Schmied et al. (2020) [45]	2019	Semi-structured interview	NS/17/12 (incl 2 new recruits)	MAge Employed = 60; Just retired = 65	Yes	Yes	T	Newly retired workers	Emotional and social support through digital coach	Implicit	Possibility of working from home and the implementation of a virtual health care coach (Sanbot Elf robot and Sola avatar).	Implicit
Habánik et al. (2021) [46]	2020–2021	Survey	Survey 1:NS/302/NA. Survey 2:NS/284/NA	AC: 18–25, 26–35, 36–45, 46–55, 56–65	Yes	No	T	Workers	Social contact with co-workers and instrumental SS for remote work	Implicit	Remote work, ICT use for work	Both implicit and explicit
Lai et al. (2021) [47]	2017–2018	Survey	265/167/NA	AC: 21–30, 31–40, 41–50	Yes	No	F, M	Workers	Employee agility and IT competency	Implicit	Enterprise Social Media, knowledge management systems, intranets, groupware, and bulletin board systems.	Explicit
Ma et al. (2021)	2018	Online survey	1500/1020/NA	AC: 55–60, 61–65, 66–70, > 70	No	No	T	Workers	Information and emotional support through social media at work	explicit	Social media usage at work	Explicit
Molino et al. (2021) [48]	NS	Focus-group and questionnaire	Qual: NS/14/NA Quant: NS/263/NA	Qual: NS. Quant: Mage = 41.44; SD = 12.01	Yes	No	T	Manufacturing workers	Supervisor SS	Explicit	Industry 4.0	Implicit
Rantanen, et al. (2021)	2019	Online survey	1128/162/NA	MAge = 43	Yes	No	T	home care workers	Informational and instrumental SS through care robots at work	Explicit	Care robots in-home care tasks	Explicit
Santini et al. (2021) [49]	2019/2021	Focus-group, telephone interview	NS/60/27	MAge: Austrian = 60.2; Italian = 60; Dutch = 65.5	Yes	Yes	F, M	Workers and Retirees	Social relationship improvement through digital technology	Explicit	Virtual coach	Explicit
Sederevičiūtė-Pačiauskienė et al. (2021) [50]	2020	in-depth interviews	NS/37/NA	AR: 19–59	Yes	No	T	Teachers	Supportive collaboration	implicit	Online teaching	Implicit
Tonnessen et al. (2021) [51]	2020	Survey	282/237/NA	AC: 30–40, 40–50, 50–60	Yes	No	F, M	Workers	Digital knowledge sharing	implicit	Teleworking	Implicit
Wrede et al. (2021) [52]	NS	Survey	1319/710/NA	MAge = 44.57; SD = 12.69	Yes	No	F, M, non-binary	Workers	SS from colleagues	Explicit	E-governement services	Implicit
Bartkowiak et al. (2022) [53]	2020/2021	in-depth interview	NS/21/18	Wave 1 AR: 31–67 Mage = 52.04; Wave 2 AR: 35–67 MAge = 58.56	Yes	No	T	Workers	Socialization and social contact	Implicit	Teleworking	Implicit
Belostecinic et al. 2022	2021	Online survey	450/377/NA	AC: 18–25, 26–40, 41–55, 55+	Yes	Yes	F, M	Workers	Employers’ informational and instrumental SS	Explicit	Teleworking	Implicit
Busch et al. 2022 [54]	NS	Survey	42/42/NS	AR: 32–66; M = 52	Yes	Yes	T	Small business workers	SS from partners	Explicit	Blended coaching format (combined face-to-face with tele-sessions, an online diary, and online courses)	Both implicit and explicit
Danieli et al. (2022) [55]	2021	Interview	60, remained 45.	MAge = 55.58; SD = 5.08	AR/AC NS	No	T	Workers	MHealth AI conversational agent at work	Explicit	TEO, Therapy Empowerment Opportunity, a mobile personal health care agent with conversational AI, mHealth app.	Explicit
De Carlo et al. (2022) [56]	2020–2021	Survey	NS/295/185	MAge = 37.6; SD: 12.3	Yes	No	F, M	Workers	Colleagues and supervisor Interpersonal support	Explicit	Teleworking	Implicit
Kim et al. (2022) [57]	2018–2019	Survey and app indicators	149/50/46	AR: 40–65 years, ET group (MAge = 47.79, SD = 7.01); ST group (MAge = 53,27, SD = 7.32)	Yes	No	F	migrant workers	SS from team leader through digital technologies	Implicit	Mobile health app based on monitoring walking Participants used a Fitbit smart watch.	Explicit
Mazzuto et al. (2022) [58]	NS	Not described	NS/8/NA	“younger” or “older” workers (born before 1980 or after)	Yes	Yes	NS	Academic workers	Training nd work support	implicit	Digital technologieswere used at work, and the participants in the education learned how to handle new techonology and safety at work	Explicit
Memon et al. (2022) [59]	2020	semi-structured interview	41/41/NA	AC: 21–30, 31–40, 41+	Yes	No	F, M,	Workers	Lack of collaboration and coordination from the supervisor	Implicit	Teleworking	Implicit
Ober (2022) [60]	2022	Survey	6000/402/NA	AC: 18–24, 25–34, 35–44, 45–54, 55+. ANALYSIS: 18–24, 25–34, 35+	Yes	No	F, M, T	Workers	Motivation to use platforms, open innovation networks, reluctance to share knowledge, and insufficient support from top management	Implicit	Open innovation platforms networks	Explicit
Scheibe et al. (2022) [61]	2021	Survey	6541/1715/NA	AC: 18–25, 26–35, 36–45, 46–55, 56+	Yes	No	F, M	Workers	Social integration	Implicit	Teleworking	Implicit
Taboroši et al. (2022) [62]	NS	Survey	NS/313/NA	AC: up to 35, 36+	Yes	No	F, M	Workers	Social networks for communication	Implicit	Social media usage in general	Explicit
Al Shamari (2022) [63]	2021	Online survey	498/262/NA	AR: 26–76 (Cohorts1946-64, 1965-80, 1981–1996)	Yes	Yes	F, M, T	Workers	Lack of relational, emotional, informational, or instrumental SS within training setting at work	Explicit	E-learning experience, working from home	Both implicit and explicit
Martínez-Pérez et al. (2023) [64]	Quant: 2018 Qual: 2020	Survey and focus-group	NS/504/NS	AR: 21–64. Mage = 37.3	Yes	No	F, M,	Workers	Lack of SS from work and lack of instrumental SS	Implicit	General ICT	Both implicit and explicit
Ferreira and Gomes (2023) [65]	2020	Survey	24,144/14,298/NA	AC: <25, 25–35, 36–50, 51–65, > 66	Yes	No	NS	Remote workers	Perceived organisational support	Explicit	Teleworking	Implicit
Lopes et al. (2023) [66]	2020	Survey	NS/573/NA	MAge = 46.8, SD = 8.10	Yes	No	T	Workers	Perceived benefits of training (e.g. better relationship with citizens, peers, and chiefs)	Implicit	Participants in digital training field group	Implicit
Oksanen et al. (2023)	2020–2022	Survey	4069/1152/656	AR: 20–66	Yes	No	F, M	Workers	Supportive working environment and SS from colleagues and supervisors	Explicit	Teleworking	Implicit
Petcu et al. (2023) [67]	2021	Survey	NS/440/NA	AC: up to 25, 26–35, 36–50, 50+	Yes	No	T	Workers	Relational communication through online tools between co-workers	implicit	Teleworking	Implicit
Raišienė et al. (2023) [68]	NS	Online survey	202/202/NA	AC: 18–24, 25–34, 35–48, 49–64	Yes	No	F, M, T	Workers	Management support	Explicit	Teleworking	Implicit
Santini et al. (2023) [69]	2021	Survey and online focus-group	91/62/NA	AR: 55+	No	No	T	Workers before and after retirement	Coach support through digital app	Explicit	Digital Coaching	Explicit
Schneider and Bousbiat (2023) [70]	2020	Survey	NS/34/NA	AC: 55–58, 59–62, 63 − 6;. MAge = 61	No	Yes	T	Workers who have retired or are about to retire	Informational support	Explicit	Smartphone and tablet usage, and the use of a robot in their daily life	Explicit
Zin et al. 2023 [51]	2022	Survey	170/170/NA	AC: 56–65, 66–75, 76–85, 86–95	No	Yes	F, M	older adults	Informational support	Implicit	smart health watch - wrist-worn wearable technologies	Explicit

Notes: NA = not applicable; NS = not stated; Quant = Quantitative; Qual = Qualitative; SS = social support; DT = digital technologies; ICT = Information and Communications Technology

*Age is reported: AC = age categories, AR = age ranges or Mage = mean age and SD = standard deviations;

**We considered older workers from the age of 45 when ”older workers” was mentioned in the study’s title (e.g. Meyers and Bagnall [71], Handley and Den Outer [72];

***Gender is reported as categories used in the analysis or results: F = female, M = male, T = total F + M

####  Participants

Regarding the number of participants invited, 19 papers did not report this information, while the remaining studies provided either precise or approximate numbers and described the invitation process. The number of participants ranged from 8 to over 14,000, depending on whether the study was qualitative, quantitative, or mixed methods. Finally, in terms of follow-up studies, only 8 studies included follow-up assessments, with participant numbers ranging from 12 to over 600, depending on the study design [35, 45, 49, 53, 56, 57, 64, 73] see Table 2).

#### Age categories used in the analysis or results of the study

Out of the 43 selected studies, 14 used age ranges (e.g., 19–63, 40–65), 13 used mean age (e.g., 42.1), and 18 used age categories (e.g., 18–25, 26–40) in the analysis. When assessing whether studies included both younger and older workers, our findings indicate that 34 studies included both, while 8 papers focussed exclusively on older workers. In one paper the age range was not stated [55] (see Table 2).

#### Type of study

Regarding the type of study, 10 studies used mixed methods, 10 studies used qualitative methods, and 28 used quantitative methods (see Table 2).

#### Type of population

Study populations include workers from various sectors such as public institutions, manufacturing, health care, academia, and other employment sectors. 17 studies included both older adults and older workers, while the remaining studies either included the general population or focussed on one specific group of workers (see Table 2).

#### Gender

21 studies analysed both men and women, two studies only women [35, 57] and one also included a category for non-binary [52]. 4 studies did not report gender in their results [38, 40, 58, 65] (see Table 2).

#### Social support (SS)

In 19 studies, SS is explicitly defined, while in the remaining 24 studies, it is implicit, i.e., SS could be inferred from one or more outcome variables. For instance, asking whether employees got on with fellow workers [39] or shared knowledge with people outside the company during the lockdown [74]. The source of support (e.g. from colleagues, supervisors, etc.) also varies across the studies (see Table 2).

#### Digital technologies (DT)

DT or technologies examined in the selected studies ranged from data entry work and teleworking to app-based interventions and ICT tools. In 20 studies, the DT is explicitly stated as part of the work or intervention, in 19 studies, its use is inferred, such as through remote work or hybrid working setups, while in the remaining 4 studies studied DT both explicitly and implicitly. For instance, Al Shamari [63] studied the experience with e-learning (an explicit DT) among Saudi Ministry of Health trainers and training coordinators who were forced to work from home (an implicit DT) during COVID-19 (see Table 2).

#### Data collection method

All studies mention a method of data collection, which included questionnaires, surveys, interviews, focus groups, or combinations of these methods. The specific data collection method employed is described for each of the 43 studies (see Table 2).

#### Countries

**Fig. 2:**
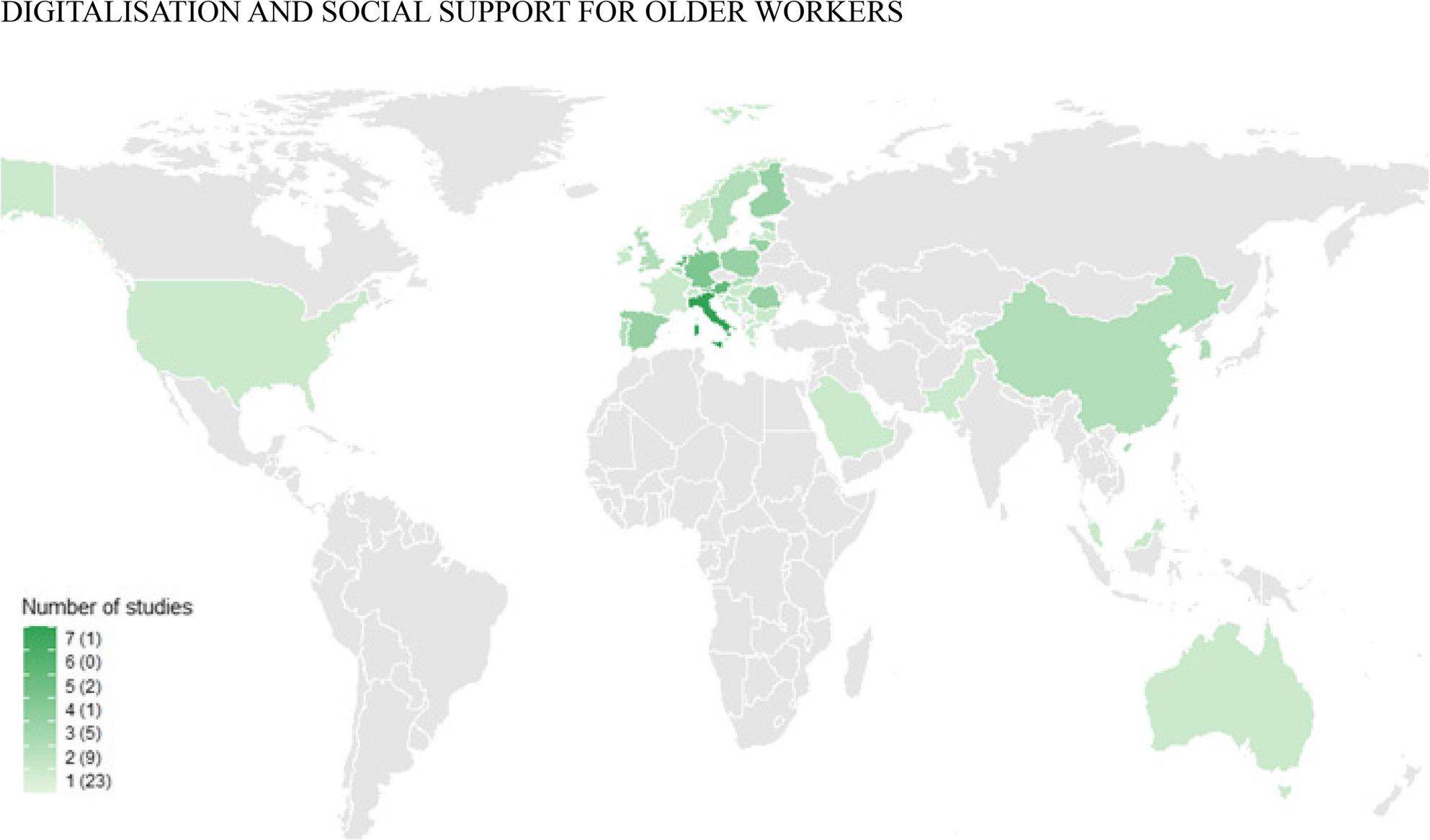
Map showing the countries where studies were conducted. The shading intensity corresponds to the frequency of studies in each country, ranging from 1 to 7, while the numbers in parentheses represents the range of appearances (e.g., only Italy appears in 7 studies)

Across the 42 studies that disclosed location, 77 countries were represented [42]. The majority of research was conducted in European countries (68 instances), followed by countries in Asian/Oceania (8) and North America (1). No relevant studies were identified from South America or Africa. Italy had the highest representation, appearing in seven studies, followed by The Netherlands and Austria, with 5 each (see Fig. 2 and Supplemental material_Countries).

### Conceptual map of social support for older workers and impact on health

The results from reviews highlight that social support is a multifaceted concept encompassing various forms of assistance that individuals may receive from others, particularly within the workplace. More important, it is often mediated using digital technologies or provided in the context of remote work. The literature spans multiple health domains, including physical health (e.g., healthy ageing and healthy activities; [49, 69], mental health (e.g., detachment; [54]; exhaustion; [54, 56], well-being [67]), social health [45, 61] and organizational outcomes (e.g., job satisfaction; 37].

Furthermore, we have identified that different studies cover one or more of the four types of online support proposed by Nick et al. [20], namely esteem/emotional support, social companionship, informational support, and instrumental support. For example, Schmied et al. [45] examined the esteem/emotional support type through the potential use of a digital coach for employees nearing retirement and retirees. Their findings indicate that individuals approaching retirement feared losing their workplace social network, and the coach provided a way to stay connected to alternative networks, thereby influencing health outcomes Similarly, Ma et al. [75] found that the direct use of social media at work enhanced both information and emotional support, as well as improved older employees’ self-efficacy at work.

Several studies illustrate instrumental support, demonstrating how supervisors or chiefs use digital technologies to support older workers in tangible ways with practical aid such as task assistance and resource provision or how they obtain this support through training [40, 42, 46, 58, 60, 63, 66, 68]. For example, Rantanenet al. [76] highlighted the role of care robots in assisting older home care workers. Employees age increases enthusiasm, but reduces self-efficacy. Lai et al. [47] highlighted employees’ agility in collaborative work is enhanced through IT competency development, enterprise social media, and knowledge management systems. Other resourceful instrumental support is health information [43, 55] and support for sustainable employability [38]. Similarly, informational support encompasses sharing knowledge, advice, or guidance [51, 57, 70, 77] with authors who discuss training and instructional content.

Relational support seems to be highlighted directly through supervisor and colleagues support [39, 48, 54, 57, 73] and communication through digital technologies [62, 67]. Another type, social companionship, reflects a sense of belonging and collaboration within the working setting [56, 65]. For example, Scheibe et al. [61] and Calderón-Gómez et al. [41] examined teamwork and social relationships at work and highlighting the relational nature of support [36, 44, 48, 50, 53, 54, 64, 69, 74]. Scheibe et al. [61] report more resilience through feeling more socially integrated in comparison to the younger employees, while Calderón-Gómez et al. [41] reported increased communicative activities although their study included very few older workers. Santini et al. [49] emphasize relational support through social relationship improvement by using digital technologies and social contact with co-workers. The AgeWell Digital Coach, a smartphone app featuring an avatar-based interface, motivational messages, and activity tracking, developed to encourage physical activity, mental well-being, and social participation during the transition to retirement, was found to improve participants’ level of self-efficacy, mental well-being, and physical activity when supplemented with human coach support. However, once the human coach stopped their involvement, this positive effects on self-efficacy and mental well-being disappeared.

However, insufficient social support may also lead to negative consequences, as attested by several studies [60, 63, 64, 72]. For instance, Memon et al. [59] found that a lack of collaboration and social interaction in remote work environments led to isolation and detachment, adversely affecting older workers’ mental health and overall well-being. Finally, indirect measures of social support are found in Meyers and Bagnall [[71];e.g. cognitive support; 2017) and Mohadis et al. [[37];e.g., social comparison and competition persuasive principles as SS).

### Digital technologies enhancing social support for older workers

Three types of digital technologies enhance social support for older workers. First of all, there are digital technologies that explicitly aim at enhancing communication, collaboration [35, 36, 38, 62] and health management [40]. For example, messaging services, social media, and video conferencing tools are used to maintain communication and coordination among colleagues, enhancing both emotional and informational support [41, 42, 75]. Additionally, online learning platforms and applications like hypermedia-based training modules provide cognitive support for older workers by facilitating independent learning and skill development [58, 71]. Other explicit digital technologies include ICT-driven health management systems, such as electronic health records and mobile health apps like the FitSenior application, which promote health-related social comparison and competition, offering persuasive encouragement through digital channels [37, 43, 55]. Secondly, teleworking setups, enabled by a range of ICT devices such as laptops, smartphones, and collaborative software [39], allow for continued collaboration and social interaction even when workers are remote [50, 52, 53, 56]. Digital technologies like care robots used in healthcare settings also fall into this category, providing informational and instrumental support for older healthcare workers [76]. Teleworking is a common theme in the studies, with multiple reports indicating that remote work setups improve workers’ social support networks through frequent interactions with colleagues over digital platforms [44, 59, 61, 67, 74]. Moreover, teleworking has been linked to increased employer-provided informational and instrumental support, enhancing older workers’ sense of social connection and their ability to access resources [65, 68, 73, 77]. Habanik et al. [46–48, 57], or indicating less support for older workers [60, 63, 64, 72]. Finally, blended formats that combine both face-to-face and digital interactions like coaching programs integrate digital platforms for mentoring, online diaries, and tele-sessions alongside traditional methods provide both emotional and instrumental support [45, 66]. For example, blended coaching programs for small business workers have been shown to enhance partner support through tele-sessions and online diaries [54]. Additionally, mobile health apps, such as the AI-powered TEO mHealth app, offer continuous conversational support, further extending the range of digital technologies available to provide support through automation [49, 51, 55, 70]. Another example is the use of digital coaching apps for retirees or workers transitioning out of the workforce, which provide emotional and informational support to facilitate smoother transitions [69].

### Theories used in studies and shaping the association between digital technologies and social support

In total, 20 studies used a theoretical foundation such as persuasive design [46], learning approaches [58], and systems approach frameworks [35]. Other studies used psychological theories with social capital [56, 57, 74, 75] or social cognitive theory [39, 57, 63] or conservation of resources theory [54, 73] or human capital theory [66] or stress theories and well-being [40, 53, 67, 68], or the theory of planned behaviour [51, 76] or behavioural change model (COM-B) [44]. Out of all, 8 studies combined theories and models, like the theory of acceptance and technology acceptance model [48, 51, 63], or applied models, such as Middleware’s model [42] or job-demands-resources (JD-R) model [48, 56, 65, 67].

In 16 studies, the concepts used were resilience [61, 67], self-efficacy and behavioural intention [39, 76], spatial intrusion [42], narrative identities [72], open innovation [60], sustainable employability [38], information processing/communication [46, 47], digital divide [41], work digitalisation [36, 77] or social support [59, 62].

Nine of the studies lacked a specific theoretical grounding [45, 49] 573; [50, 52, 55, 64, 70, 71].

### Instruments used for measuring social support

The results regarding social support for older employees’ use of digital technology are measured with different types of instruments in the different studies, i.e. the design of the measuring instruments was different. Among the studies, 12 studies used interviews [43, 45, 48–50, 53, 55, 59, 64, 69, 71, 72], and 30 studies used questions and questionnaires to measure social support for older workers’ use of digital technology [35, 36, 38–40, 42, 46, 47, 52, 55–58, 60, 62–65, 73–76]– [41, 51, 66–70, 77]. One study [61] measured frequent use of social support for digital technologies with the intention of measuring social support in older workers’ use of digital technology.

### Instruments used for measuring the digital technologies

The digital technology used by the older employees differed. However, not all studies measured the older employees’ attitude, experience or frequency in using digital technology. In total, 20 studies used questions and questionnaires to measure the older employees’ experiences and attitudes towards the digital technology [35, 38, 39, 41, 42, 46–48, 60, 62, 64–68, 73, 75, 76], [51, 70],. There were six studies that conducted interviews with the older employees to investigate their experiences and attitudes towards the digital technology [36, 45, 49, 53, 59, 64]. In six studies, measurements were made to investigate and estimate how often and for how long the older employees used the digital technology [36, 50, 57, 59, 71, 74].

## Discussion

The scoping review analysed the role of digital technologies in providing social support to older workers, aiming to determine which technologies are most used and their impacts on social support and health and well-being. Notably, most of the studies we reviewed collected data in 2020 or later, coinciding with the COVID-19 pandemic and its aftermath. This timing likely influenced the findings: the pandemic’s rapid shift toward remote work and greater reliance on digital communication tools underscored the need for online forms of social support for employees. Our discussion reflects on the dual impacts of these digital technologies, both positive and negative, on older workers’ social support and health, and situates these findings within existing theoretical frameworks.

### Digital technologies shaping social support and health for older workers

With the rise of digital technologies, the delivery of social support has evolved in ways that align with and extend existing theoretical perspectives. From the standpoint of Lakey and Cohen’s [15] stress and coping and relational approaches, digital technologies such as teleworking platforms, ICT systems, and mobile health applications introduce new mechanisms through which both explicit and implicit forms of support can emerge. Explicit relational support in the reviewed studies was delivered through structured digital interventions such as coaching programs and mobile health apps [57, 69, 76]. At the same time, this review shows that digital technologies also create bottom-up, implicit support pathways, particularly in remote work settings. Remote working environments, often challenging for older workers with lower digital skills, can nonetheless promote social integration as workers use communication technologies to remain connected and exchange knowledge [61, 74]. These findings suggest that digital settings can mediate bottom-up, implicit pathways for social support and that older workers actively appropriate digital platforms to access emotional, social companionship, informational, and instrumental support, as described by Nick et al. [20]. This pattern indicates that digital contexts may expand traditional models of social support by revealing how support can arise indirectly through everyday digital interactions. 

### Positive effects on social support

Digital technologies have become essential in providing social support to older workers across different contexts and industries. Across the reviewed studies, digital tools ranged from basic communication platforms to more sophisticated systems designed to facilitate emotional support, social companionship, informational support, and instrumental assistance [35, 44, 61, 69, 76], reflecting the types of support outlined by Nick et al. [20]. The findings reported in this scoping review provide evidence that explicit and blended forms of support, such as digital coaching programs, mobile health applications, and mixed face-to-face/online formats, can address the emotional and practical needs of older workers, particularly those transitioning out of the workforce, with support often coming from colleagues and supervisors [34, 54, 65, 69]. The studies also highlight the importance of organisational and supervisor support in enabling older workers to adopt new technologies and maintain work engagement [47, 48, 77]. Conversely, insufficient organisational support can undermine the effectiveness and sustainability of these digital tools for older employees [77]. In addition, the review indicates that digitally mediated social support from outside the workplace (e.g., support from partners or family during periods of remote work) warrants consideration, given its positive effects on goal achievement and stress reduction [59]. In summary, digital technologies in the workplace provide multiple forms of social support, ranging from explicit, structured interventions such as mobile apps and digital coaches to more implicit support embedded in remote work and communication platforms.

### Challenges and negative implications

Despite these benefits, several studies reveal challenges. Older workers often face a digital divide, where their digital skill levels do not always align with job requirements, leading to feelings of isolation or exclusion. For instance, in teleworking environments, many older employees reported feelings of social deprivation and mental exhaustion, particularly when lacking peer or supervisory support [52, 68]. Studies also noted that older employees working from home encountered reduced interaction with peers and supervisors, which can decrease job satisfaction and mental well-being [59, 73]. Furthermore, teleworking and remote work setups can create a sense of isolation if not managed properly, with some workers reporting reduced collaboration and limited interaction with supervisors [59]. This detachment can lead to decreased social companionship, a critical component of social support, and can negatively impact employee morale and well-being. Nevertheless, the flexibility and connectivity afforded by digital technologies have the potential to greatly enhance support, particularly when combined with human interactions [45, 54].

Many older workers express a need for additional training to navigate new digital platforms effectively. Findings by Mazzuto et al. [58] reveal a discrepancy in learning rates, indicating that older workers often require more times and support to adapt to evolving technological tools. This suggests that tailored training programs could play a crucial role in enhancing digital adaptability and sustaining employability among older workers.

The literature also highlights divergent outcomes regarding the well-being of older workers. These outcomes appear to be shaped by several factors, such as presence or absence of organizational support [6, 65], the digital literacy level of older workers, the type of digital technologies employed [78] and the broader implementation. Accordingly, the impact of digital technologies on older workers is not unidimensional but multidimensional, encompassing social, organizational and individual layers.

## Limits of the study

Our study also has several limitations. First, the search was confined to English-language publications. This exclusion may limit the comprehensiveness and generalizability of our findings, as studies published in other languages could provide alternative perspectives or results. The exclusion of non-peer-reviewed sources represents an additional limitation, as grey literature may contain relevant data or studies with non-significant results that are not captured in the published record. Moreover, this review is predominantly based on research conducted in developed countries. The relationships identified may therefore be contingent upon specific contextual factors, e.g., cultural norms that differ in developing nations. Future research is thus needed to validate these findings across a broader range of geographical and economic settings.

The studies included also reflect considerable variability in designs. However, the dominance of European contexts in the available literature may further limit the generalizability of the findings to other cultural and policy settings. In addition, some studies did not exclusively examine older workers but included broader working populations, which may have diluted age-specific insights.

Finally, the absence of standardized instruments for measuring social support remains a significant gap, and addressing this issue should be considered a priority for future research. Lastly, the conclusions drawn from this review should be interpreted with caution, as several included studies were of only moderate methodological quality.

## Conclusion

The article highlights the dualistic impact of digitalisation on social support for older workers, highlighting both opportunities and challenges within modern workplaces. On the positive side, digital technologies enable older workers to receive various forms of social support (e.g., esteem/emotional support, social companionship, informational support, and instrumental support) through platforms that promote communication, such as teleworking, enterprise social media, and health-related apps. These technologies facilitate professional connections and provide mental health benefits, often fostering a sense of inclusion and continuous learning and are aligned with Nick and colleagues’ perspective [20] regarding types of online support. However, digitalisation also introduces challenges, especially for those with limited digital skills, potentially leading to social isolation, technostress, and a feeling of inadequacy in fast-paced digital environments. The reviewed studies highlight that, although digital technologies can bolster older workers’ productivity and connectivity, the benefits are contingent on adequate training and organisational support. The research thus points to the need for targeted digital literacy programs and sustained managerial backing to mitigate negative impacts, ensuring that digitalisation enhances rather than hinders the well-being of older employees.

Results also reflect the lens of Lakey and Cohen’s [15] three theoretical perspectives on social support. Regarding the stress and coping approach, results indicate that digital technologies such as teleworking platforms and communication apps provide implicit support, reducing the stress of isolation in remote work environments. However, older workers also report stress related to the use of technologies and a digital divide, implying that while these technologies can offer stress relief through connection, they can also introduce stress due to the necessity of digital skill adaptation. Tailored digital literacy programs could mitigate this stress, enabling these technologies to fulfil a more effective stress-buffering role. This approach is also supported by recent study findings that show metaverse could be used to enhance social support because it can offer simulated social situations to practice and improve social interaction skills [79]. The constructionist approach aligns with the findings in the article that digital technologies provide emotional support and self-efficacy for older workers. For instance, apps that offer digital coaching or feedback boost a sense of autonomy and competence, enhancing well-being independent of stress levels. The development of skills and encouragement through technologies like mobile health apps also reinforces self-esteem, aiding workers in managing their roles effectively, even amid digital transitions.

Finally, the relational approach is illustrated using digital technologies such as enterprise social media, which foster relational support by promising communication and collaboration. Relationships formed or maintained through remote platforms enable emotional and informational exchanges that are essential for social companionship. However, insufficient interaction in remote settings can also leads to isolation, signalling the need to balance digital and in-person interactions to foster low-conflict, meaningful connections that enhance well-being.

In conclusion, digital technologies are reshaping the landscape of social support by offering both implicit and explicit mechanisms for delivering emotional, informational, and instrumental support. When effectively integrated, these technologies enhance communication, collaboration, and social integration among older workers, thereby contributing to improved work experiences and overall well-being [35, 61, 75].

## Supplementary Information

Supplementary Material 1.

Supplementary Material 2.

Supplementary Material 3.

## Data Availability

Not applicable.

## References

[1] OECD. Pensions at a glance 2023 OECD. 2023. https://www.oecd.org/en/publications/pensions-at-a-glance-2023_678055dd-en.html. Accessed 26 Nov 2024.

[2] Green A. Artificial intelligence and the changing demand for skills in the labour market. Paris: OECD Publication; 2024.

[3] WHO. Decade of healthy ageing (2020–2030) WHO. 2022. https://www.who.int/initiatives/decade-of-healthy-ageing. Accessed 26 Nov 2024.

[4] Eurostat. Ageing Europe - statistics on working and moving into retirement Eurostat. 2024. https://ec.europa.eu/eurostat/statistics-explained/index.php?title=Ageing_Europe_-_statistics_on_working_and_moving_into_retirement. Accessed 26 May 2024.

[5] Decade of Healthy Ageing. 2021–2030 | Division for Inclusive Social Development (DISD). Un.org. 2021 [cited 2024 Nov 27]. Available from: https://social.desa.un.org/sdn/decade-of-healthy-ageing-2021-2030. Accessed 26 Nov 2024.

[6] Nilsson K, Nilsson E. Organisational measures and strategies foeder a healthy and sustainable extended working life and employability-a deductive content analysis with data including employees, first line managers, trade union representatives and HR-practitioners. Int J Environ Res Public Health. 2021;18:5626. 10.3390/ijerph18115626. 34070299 10.3390/ijerph18115626 PMC8197545

[7] Spijker JJ, Barlın H, Dritsaki M, Gu Y, Klavina A, Yaylagul NK, Tofan CM. (2025). The impact of digital technology on the physical health of older workers. Scoping Rev JMIR Aging, 8(1) e78406 10.2196/78406 PMC12673309 41253363

[8] Marvell R, Cox A, Institute for Employment Studies. What do older workers value about work and why? Brighton (UK): Centre for Ageing Better & ; 2017. Available from: https://ageing-better.org.uk/sites/default/files/2017-12/What-do-older-workers-value.pdf

[9] Crawford JO, Graveling RA, Cowie HA, Dixon K. The health safety and health promotion needs of older workers. Occup Med (Lond). 2010;60(3):184–92. 20423949 10.1093/occmed/kqq028

[10] McCarthy J, Heraty N, Cross C, Cleveland JN. Who is considered an ‘older worker’? Extending our conceptualisation of ‘older’ from an organisational decision maker perspective. Hum Resour Manage J. 2014;24(4):374–93. 10.1111/1748-8583.12041.

[11] Nilsson K. A sustainable working life for all ages - the swAge-model. Appl Ergon. 2020;86:103082. 10.1016/j.apergo.2020.103082. 32342898 10.1016/j.apergo.2020.103082

[12] Carneiro LL, Silva IJ. Demands and resources in work mediated by digital labour platforms: A scoping review. OS – Revista De Ciências Sociais. 2023;123. 10.1590/1984-92302023v30n0004EN.

[13] Turner RJ. Social support as a contingency in psychological well-being. J Health Soc Behav. 1981;22:357–67. 10.2307/2136677.

[14] Cobb S. 1976. Social support as a moderator of life stress. Psychosom Med. 1976;38:300–14. 10.1097/00006842-197609000-00003 10.1097/00006842-197609000-00003 981490

[15] Lakey B, Cohen S. Social support theory and measurement. In: Cohen S, Underwood LG, Gottlieb BH, editors. Social support measurement and intervention: a guide for health and social scientists. Oxford: Oxford University Press; 2000. p. 29–52.

[16] LaMeres BJ. Introduction: analog vs. digital. In: LaMeres BJ, editor. Introduction to logic circuits & logic design with verilog. Cham: Springer International Publishing; 2017. p. 1–5.

[17] Quan-Haase A, Mo GY, Wellman B. Connected seniors: how older adults in East York exchange social support online and offline. Inf Commun Soc. 2017;20:967–83. 10.1080/1369118X.2017.1305428.

[18] Nimrod G. Technostress: measuring a new threat to well-being in later life. Aging Ment Health. 2018;22:1080–7. 10.1080/13607863.2017.1334037. 28562064 10.1080/13607863.2017.1334037

[19] Alcover CM, Guglielmi D, Depolo M, Mazzetti G. Aging-and-tech job vulnerability: a proposed framework on the dual impact of aging and AI, robotics, and automation among older workers. Organ Psychol Rev. 2021;11:175–201. 10.1177/2041386621992105.

[20] Nick EA, Cole DA, Cho SJ, Smith DK, Carter TG, Zelkowitz RL. The online social support scale: measure development and validation. Psychol Assess. 2018;30:1127–43. 10.1037/pas0000558. 29781664 10.1037/pas0000558 PMC6107390

[21] Czaja SJ, Ceruso M. The promise of artificial intelligence in supporting an aging population. J Cogn Eng Decis Mak. 2022;16:182–93. 10.1177/15553434221129914.

[22] Dare J, Green L. Rethinking social support in women’s midlife years: women’s experiences of social support in online environments. Eur J Cult Stud. 2011;14:473–90. 10.1177/1367549411412203.

[23] Francis J, Kadylak T, Makki TW, Rikard RV, Cotten SR. Catalyst to connection: when technical difficulties lead to social support for older adults. Am Behav Sci. 2018;62:1167–85. 10.1177/0002764218773829.

[24] Mendel P, O’Hora J, Zhang L, Stockdale S, Dixon EL, Gilmore J, Jones F, Jones A, Williams P, Sharif MZ, et al. Engaging community networks to improve depression services: A cluster-randomized trial of a community engagement and planning intervention. Community Ment Health J. 2021;57:457–69. 10.1007/s10597-020-00632-5. 32430557 10.1007/s10597-020-00632-5 PMC7906961

[25] Marston HR, Musselwhite CBA. Improving older people’s lives through digital technology and practices. Gerontol Geriatr Med. 2021;7:23337214211036255. 10.1177/23337214211036255. 34527764 10.1177/23337214211036255 PMC8436304

[26] Utz S, Breuer J. The relationship between use of social network sites, online social support, and well-being: results from a six-wave longitudinal study. J Media Psychol. 2017;29:115–25. 10.1027/1864-1105/a000222. 29147141 10.1027/1864-1105/a000222 PMC5683734

[27] Thompson LF, Atkins SG. Technology, mobility, and poverty reduction. In: Carr SC, editor. The psychology of global mobility. New York: Springer; 2010. pp. 301–22.

[28] Thompson LF, Mayhorn CB. Aging workers and technology. In: Borman WC, Hedge JW, editors. The Oxford handbook of work and aging. Oxford: Oxford University Press; 2012. pp. 341–60.

[29] Tajfel H. Social identity and intergroup relations. New York: Cambridge University Press; 1982.

[30] Tixier M, Lewkowicz M. Designing social support online services for communities of family caregivers. In: Abramowicz W, Flejter D, editors. Business information systems workshops. Cham: Springer; 2009. pp. 336–47.

[31] Salehi S, Abedi A, Balakrishnan S, Gholamrezanezhad A. Coronavirus disease 2019 (COVID-19): a systematic review of imaging findings in 919 patients. AJR Am J Roentgenol. 2020;215:87–93. 10.2214/ajr.20.23034. 32174129 10.2214/AJR.20.23034

[32] Peters MDJ, Marnie C, Tricco AC, Pollock D, Munn Z, Alexander L, McInerney P, Godfrey CM, Khalil H. Updated methodological guidance for the conduct of scoping reviews. JBI Evid Synth. 2020;18:2119–26. 10.11124/jbies-20-00167. 33038124 10.11124/JBIES-20-00167

[33] Tetzlaff J, Page M, Moher D. PNS154 the PRISMA 2020 statement: development of and key changes in an updated guideline for reporting systematic reviews and meta-analyses. Value Heal. 2020;23:S312–3. 10.1016/J.JVAL.2020.04.1154.

[34] Hong QN, Pluye P, Fàbregues S, Bartlett G, Boardman F, Cargo M, et al. Improving the content validity of the mixed methods appraisal tool: a modified e-Delphi study. J Clin Epidemiol. 2019;111:49-59.e1. 10.1016/j.jclinepi.2019.03.008. 30905698 10.1016/j.jclinepi.2019.03.008

[35] Aborg C, Fernström E, Ericson MO. Work content and satisfaction before and after a reorganisation of data entry work. Appl Ergon. 1998;29:473–80. 10.1016/s0003-6870(98)00009-x. 9796793 10.1016/s0003-6870(98)00009-x

[36] Carayon P, Karsh BT. Sociotechnical issues in the implementation of imaging technology. Behav Inf Technol. 2000;19:247–62. 10.1080/01449290050086363.

[37] Mohadis HM, Mohamad Ali N, Smeaton AF. Designing a persuasive physical activity application for older workers: understanding end-user perceptions. Behav Inf Technol. 2016;35:1102–14. 10.1080/0144929X.2016.1211737.

[38] Verbrugghe M, Kuipers Y, Vriesacker B, Peeters I, Mortelmans K. Sustainable employability for older workers: an explorative survey of belgian companies. Arch Public Health. 2016;74:15. 10.1186/s13690-016-0128-x. 27127626 10.1186/s13690-016-0128-x PMC4848867

[39] Arvola R, Tint P, Kristjuhan U, Siirak V. Impact of telework on the perceived work environment of older workers. Sci Ann Econ Bus. 2017;64:199–214. 10.1515/SAEB-2017-0013.

[40] Hauk N, Göritz AS, Krumm S. The mediating role of coping behavior on the age-technostress relationship: a longitudinal multilevel mediation model. PLoS One. 2019;14:e0213349. 10.1371/journal.pone.0213349. 30835773 10.1371/journal.pone.0213349 PMC6400396

[41] Calderón-Gómez D, Casas-Mas B, Urraco-Solanilla M, Revilla JC. The labour digital divide: digital dimensions of labour market segmentation. Work Organ Labour Glob. 2020;14:7–30. 10.13169/WORKORGALABOGLOB.14.2.0007.

[42] Chandra S, Shirish A, Srivastava SC. Theorizing technological spatial intrusion for ICT enabled employee innovation: the mediating role of perceived usefulness. Technol Forecast Soc Change. 2020;161:120320. 10.1016/J.TECHFORE.2020.120320.

[43] De Leeuw JA, Woltjer H, Kool RB. Identification of factors influencing the adoption of health information technology by nurses who are digitally lagging: in-depth interview study. J Med Internet Res. 2020;22:e15630. 10.2196/15630. 32663142 10.2196/15630 PMC7455866

[44] Middleton M, Somerset S, Evans C, Blake H. Test@Work texts: mobile phone messaging to increase awareness of HIV and HIV testing in UK construction employees during the COVID-19 pandemic. Int J Environ Res Public Health. 2020;17:7819. 10.3390/ijerph17217819. 33114546 10.3390/ijerph17217819 PMC7672579

[45] Schmied M, Igerc I, Schneider C. A digital health coach for younger seniors - user centred requirements collection. Stud Health Technol Inform. 2020;271:137–44. 10.3233/shti200089. 32578556 10.3233/SHTI200089

[46] Habánik J, Grenčíková A, Šrámka M, Húževka M. Changes in the organization of work under the influence of COVID-19 pandemic and industry 4.0. Econ Sociol. 2021;14:228–41. 10.14254/2071-789X.2021/14-4/13.

[47] Lai H, Pitafi AH, Hasany N, Islam T. Enhancing employee agility through information technology competency: an empirical study of China. SAGE Open. 2021. 10.1177/21582440211006687.

[48] Molino M, Cortese CG, Ghislieri C. Technology acceptance and leadership 4.0: a quali-quantitative study. Int J Environ Res Public Health. 2021;18:10845. 10.3390/ijerph182010845. 34682588 10.3390/ijerph182010845 PMC8535315

[49] Santini S, Stara V, Galassi F, Merizzi A, Schneider C, Schwammer S, et al. User requirements analysis of an embodied conversational agent for coaching older adults to choose active and healthy ageing behaviors during the transition to retirement: a cross-national user centered design study. Int J Environ Res Public Health. 2021;18:9681. 10.3390/ijerph18189681. 34574615 10.3390/ijerph18189681 PMC8468148

[50] Sederevičiūtė-Păciauskienė Ž, Valantinaitė I, Kliukas R. Communion, care, and leadership in computer-mediated learning during the early stage of COVID-19. Sustainability. 2021;13:4234. 10.3390/SU13084234.

[51] Zin KSLT, Kim S, Kim HS, Feyissa IF. A study on technology acceptance of digital healthcare among older Korean adults using extended tam (extended technology acceptance model). Adm Sci. 2023;13:42. 10.3390/ADMSCI13020042.

[52] Wrede SJS, Rodil Dos Anjos D, Kettschau JP, Broding HC, Claassen K. Risk factors for digital stress in German public administrations. BMC Public Health. 2021;21:2204. 10.1186/s12889-021-12247-w. 34856964 10.1186/s12889-021-12247-w PMC8639295

[53] Bartkowiak G, Krugiełka A, Dama S, Kostrzewa-Demczuk P, Gaweł-Luty E. Academic teachers about their productivity and a sense of well-being in the current COVID-19 epidemic. Int J Environ Res Public Health. 2022;19:4970. 10.3390/ijerph19094970. 35564364 10.3390/ijerph19094970 PMC9100625

[54] Busch C, Dreyer R, Janneck M. Blended health coaching for work-linked couples: coaches’ intervention fidelity and empathy matter! Coach Theor Prax. 2022;8:43–58. 10.1365/S40896-022-00065-9.

[55] Danieli M, Ciulli T, Mousavi SM, Silvestri G, Barbato S, Di Natale L, et al. Assessing the impact of conversational artificial intelligence in the treatment of stress and anxiety in aging adults: randomized controlled trial. JMIR Ment Health. 2022;9:e38067. 10.2196/38067. 36149730 10.2196/38067 PMC9547337

[56] De Carlo A, Girardi D, Dal Corso L, Arcucci E, Falco A. Out of sight, out of mind? A longitudinal investigation of smart working and burnout in the context of the job demands–resources model during the COVID-19 pandemic. Sustainability. 2022;14:7121. 10.3390/SU14127121.

[57] Kim Y, Lee H, Chung ML. Living labs for a mobile app-based health program: effectiveness of a 24-week walking intervention for cardiovascular disease risk reduction among female Korean-Chinese migrant workers: a randomized controlled trial. Arch Public Health. 2022;80:181. 10.1186/s13690-022-00941-z. 35927769 10.1186/s13690-022-00941-z PMC9351079

[58] Mazzuto G, Antomarioni S, Marcucci G, Ciarapica FE, Bevilacqua M. Learning-by-doing safety and maintenance practices: a pilot course. Sustainability. 2022;14:9635. 10.3390/SU14159635.

[59] Memon MA, Shaikh S, Mirza MZ, Obaid A, Muenjohn N, Ting H. Work-from-home in the new normal: a phenomenological inquiry into employees’ mental health. Int J Environ Res Public Health. 2022;20:48. 10.3390/ijerph20010048. 36612370 10.3390/ijerph20010048 PMC9819185

[60] Ober J. Open innovation in the ICT industry: substantiation from Poland. J Open Innov Technol Mark Complex. 2022;8:158. 10.3390/JOITMC8030158.

[61] Scheibe S, De Bloom J, Modderman T. Resilience during crisis and the role of age: involuntary telework during the COVID-19 pandemic. Int J Environ Res Public Health. 2022;19:1762. 10.3390/ijerph19031762. 35162785 10.3390/ijerph19031762 PMC8834860

[62] Taboroši S, Popović J, Poštin J, Rajković J, Berber N, Nikolić M. Impact of using social media networks on individual work-related outcomes. Sustainability. 2022;14:7646. 10.3390/SU14137646.

[63] Al Shamari D. Challenges and barriers to e-learning experienced by trainers and training coordinators in the Ministry of Health in Saudi Arabia during the COVID-19 crisis. PLoS One. 2022;17:e0274816. 10.1371/journal.pone.0274816. 36251639 10.1371/journal.pone.0274816 PMC9576076

[64] Martínez-Pérez A, Lezcano-Barbero F, Zabaleta-González R, Casado-Muñoz R. Usage of ICT among social educators—an analysis of current practice in Spain. Educ Sci. 2023;13:231. 10.3390/EDUCSCI13030231.

[65] Ferreira P, Gomes S. Work–life balance and work from home experience: perceived organizational support and resilience of European workers during COVID-19. Adm Sci. 2023;13:153. 10.3390/ADMSCI13060153.

[66] Lopes AS, Sargento A, Farto J. Training in digital skills—the perspective of workers in public sector. Sustainability. 2023;15:577. 10.3390/SU151310577.

[67] Petcu MA, Sobolevschi-David MI, Crețu RF, Curea SC, Hristea AM, Oancea-Negescu MD, et al. Telework: a social and emotional perspective of the impact on employees’ wellbeing in the COVID-19 pandemic. Int J Environ Res Public Health. 2023;20:1811. 10.3390/ijerph20031811. 36767179 10.3390/ijerph20031811 PMC9914358

[68] Raišienė AG, Danauskė E, Kavaliauskienė K, Gudžinskienė V. Occupational stress-induced consequences to employees in the context of teleworking from home: a preliminary study. Adm Sci. 2023;13:55. 10.3390/ADMSCI13020055.

[69] Santini S, Fabbietti P, Galassi F, Merizzi A, Kropf J, Hungerländer N, et al. The impact of digital coaching intervention for improving healthy ageing dimensions among older adults during their transition from work to retirement. Int J Environ Res Public Health. 2023;20:4034. 10.3390/ijerph20054034. 36901045 10.3390/ijerph20054034 PMC10001821

[70] Schneider C, Bousbiat H. Coaching robots for older seniors: do they get what they expect? Insights from an Austrian study. Int J Environ Res Public Health. 2023;20:2965. 10.3390/ijerph20042965. 36833659 10.3390/ijerph20042965 PMC9963592

[71] Meyers CA, Bagnall RG. The challenges of undergraduate online learning experienced by older workers in career transition. Int J Lifelong Educ. 2017;36:442–57. 10.1080/02601370.2016.1276107.

[72] Handley K, Den Outer B. Narrating ‘potential’: older knowledge workers’ anticipatory narratives about their future employment. Ageing Soc. 2021;41:2375–95. 10.1017/S0144686X20000252.

[73] Oksanen A, Oksa R, Celuch M, Cvetkovic A, Savolainen I. COVID-19 anxiety and wellbeing at work in Finland during 2020–2022: a 5-wave longitudinal survey study. Int J Environ Res Public Health. 2022;20:680. 10.3390/ijerph20010680. 36612998 10.3390/ijerph20010680 PMC9819787

[74] Tønnessen Ø, Dhir A, Flåten BT. Digital knowledge sharing and creative performance: work from home during the COVID-19 pandemic. Technol Forecast Soc Change. 2021;170:120866. 10.1016/j.techfore.2021.120866. 35068596 10.1016/j.techfore.2021.120866 PMC8764621

[75] Ma Y, Liang C, Gu D, Zhao S, Yang X, Wang X. How social media use at work affects improvement of older people’s willingness to delay retirement during transfer from demographic bonus to health bonus: causal relationship empirical study. J Med Internet Res. 2021;23:e18264. 10.2196/18264. 33565983 10.2196/18264 PMC7904398

[76] Rantanen T, Leppälahti T, Coco K. The introduction of care robots as a leadership challenge in home care facilities in Finland. Nurs Open. 2022;9:1854–64. 10.1002/nop2.933. 34110103 10.1002/nop2.933 PMC8994953

[77] Belostecinic G, Mogoș RI, Popescu ML, Burlacu S, Rădulescu CV, Bodislav DA, et al. Teleworking-an economic and social impact during COVID-19 pandemic: a data mining analysis. Int J Environ Res Public Health. 2021;19:298. 10.3390/ijerph19010298. 35010555 10.3390/ijerph19010298 PMC8751029

[78] Nedeljko M, Gu Y, Bostan CM. The dual impact of technological tools on health and technostress among older workers: an integrative literature review. Cogn Tech Work. 2024;26:47–61. 10.1007/s10111-023-00741-7.

[79] Buragohain D, Khichar S, Deng C, Meng Y, Chaudhary S. Analyzing metaverse-based digital therapies, their effectiveness, and potential risks in mental healthcare. Sci Rep. 2025;15(1):17066. 10.1038/s41598-025-00916-4. 40379748 10.1038/s41598-025-00916-4 PMC12084641

