# Supplementary material for: Redefining social support: a scoping review of the effects of digital technologies on the social support of older workers
Source: BMC Public Health. 2026 Jan 14;26:542. doi: 10.1186/s12889-025-26155-w (PMC12888202; doi:10.1186/s12889-025-26155-w)
Supplement: Supplementary file 1 — Supplementary Material 1. [file 12889_2025_26155_MOESM1_ESM.docx]

Multimedia Appendix 4. PRISMA-ScR checklist.

Table of contents

[Study details according to the PRISMA-ScR checklist [49]. 2](#_heading=h.m53lsiu11fj1)

[Item 1. Title 2](#_heading=h.z4b5dnrippt3)

[**Abstract** 2](#_heading=h.4yq4vf4766a5)

[Item 2. Structured summary 2](#_heading=h.lhthw0yegf7q)

[Item 3. Rationale 2](#_heading=h.8mcgfz3ykva)

[Item 4. Objectives 2](#_heading=h.aia3kyl0p40)

[**Methods** 3](#_heading=h.dvifqi2o9nft)

[Item 5. Protocol and registration 3](#_heading=h.nf574prr12me)

[Item 6. Eligibility criteria 3](#_heading=h.egzjx8gcmvjf)

[Item 7. Information sources 3](#_heading=h.xikoyzqp4hm)

[Item 8. Search 3](#_heading=h.m04ld01urjxy)

[Item 9. Selection of sources of evidence 3](#_heading=h.tlj40ze5yt0n)

[Item 10. Data charting process 4](#_heading=h.piygbvrg0v6v)

[Item 11. Data items 4](#_heading=h.mbuu0h7rhjkf)

[Item 12. Critical appraisal of individual sources of evidence 5](#_heading=h.kwkw91on9ltm)

[Item 13. Summary measures 5](#_heading=h.b6h422bo8404)

[Item 14. Synthesis of results 5](#_heading=h.txkxge8z7x9s)

[Item 15. Risk of bias across studies 5](#_heading=h.4kzwzux4bhns)

[Item 16. Additional analyses 5](#_heading=h.hm9jiq3uapde)

[**Results** 5](#_heading=h.hn4tydm74z4k)

[Item 17. Selection of sources of evidence 5](#_heading=h.mqdpak3zgii6)

[Item 18 Characteristics of sources of evidence 5](#_heading=h.m4ij21tv9rwo)

[Item 19 Critical appraisal within sources of evidence 5](#_heading=h.uu5l1l4c2myu)

[Item 20 Results of individual sources of evidence 5](#_heading=h.lhpj5fu912gu)

[Item 21 Synthesis of results 5](#_heading=h.pnv85lqm94cn)

[Item 22 Risk of bias across studies 5](#_heading=h.nhroqv40xe7p)

[Item 23 Additional analyses 5](#_heading=h.lz5potypevi5)

[**Discussion** 6](#_heading=h.y1kuc33mvx0c)

[Item 24 Summary of evidence 6](#_heading=h.jhzdlgdors37)

[Item 27: Funding 6](#_heading=h.k1pt83p6e4e2)

[References 6](#_heading=h.cnet5mviidos)

# Study details according to the PRISMA-ScR checklist [49].

## Item 1. Title

Redefining Social Support: The Effect of Digital Technologies on the Social Support of Older Workers. A Scoping Review

## **Abstract**

## Item 2. Structured summary

**Background:** The rapid digitalisation of workplaces presents both challenges and opportunities for older workers. This scoping review examines how digital technologies impact social support for older workers, focusing on emotional, informational, and instrumental support within professional environments. While social support is critical for well-being and productivity in ageing workforces, the effects of digitalisation on social support dynamics remain insufficiently understood.

**Objective:** This scoping review aims to understand the role of digital technology in enhancing social support for older workers.

**Methods:** Following Joanna Briggs Institute and PRISMA-ScR guidelines, a comprehensive search strategy was conducted across databases like ERIH, Web of Science, Scopus, and PubMed from anytime to 2023 to identify peer-reviewed studies involving digital technologies used by older workers, generally considered as workers aged 50 years or older. Covidence software facilitated the screening of over 5000 scientific papers, study selection, and data extraction, and the Mixed Methods Appraisal Tool (MMAT) assessed quality. Findings were synthesized through descriptive statistics and narrative analysis.

**Results:** Forty-three studies met inclusion criteria. Digital technologies were found to enhance various forms of social support in both explicit and implicit ways. Remote work tools, messaging apps, and telemedicine platforms facilitated emotional connection and informational exchange. However, digitalisation also introduced barriers. Some older workers reported isolation, reduced informal contact, and technostress, underscoring disparities in digital literacy and adaptation.

**Conclusions:** Digitalisation exerts a dual impact on social support for older workers: it can strengthen professional connectedness yet also heighten vulnerability to stress and exclusion. Targeted digital literacy initiatives and sustained managerial engagement are crucial to ensure that technology enhances, rather than undermines, well-being and productivity among ageing employees.

**Introduction**

## Item 3. Rationale

The aim of this scoping review is to locate and synthesize evidence on the role of digital technology in enhancing social support for older workers.

## Item 4. Objectives

This scoping review aims to examine and summarize the scientific evidence on how digital technology enhance social support for older workers, focusing on the following topics:

1) Study design and focus.

2) Digital technology type

3) Social support type

4) Outcomes

5) Evidence gaps in this field

## **Methods**

## Item 5. Protocol and registration

Protocol of the scoping review for registration was not developed.

## Item 6. Eligibility criteria

The eligibility criteria for this scoping review are based on the Population, Concept and Context (PCC) criteria (see also Textbox 1 in main text):

Inclusion criteria

1. Population: older workers (50+ included in study)
2. Concept: digital technologies related to social support at workplace
3. Context: workplace contexts
4. Setting: nonclinical
5. Study type: original studies with any design or data type (quantitative and qualitative)
6. Publication status: published in a peer-reviewed journal
7. Publication language: English
8. Full-text available

Exclusion criteria:

1. Population: younger workers (50+ not included in study)
2. Concept: analogue technology used at work for social support
3. Context: not associated with employment and social support
4. Setting: clinical
5. Study type: other study types (e.g. protocols, narrative reviews or systematic reviews)
6. Publication status: published without peer-review, dissertations, books, conference papers, letters, editorials.
7. Publication language: written in a language other than English
8. Full-text not available

## Item 7. Information sources

The information sources for this scoping review include the following international bibliographic databases: ERIH, Web of Science, Scopus, PubMed, PsycINFO, Proquest on 04 May 2023. For this scoping review, the databases PubMed, PROSPERO, and JBI Evidence Synthesis were searched on June and July, 2023; however, no current or ongoing systematic or scoping reviews related to the topic of interest were identified. A preliminary search was also conducted around the same time using the databases MEDLINE, Cochrane, and Epistemonikos.

## Item 8. Search

The syntax for the electronic search was developed and calibrated throughout March-April 2023 within the research team. The full search strategy is reported in the scoping review.

## Item 9. Selection of sources of evidence

After importing references and removing duplicates on COVIDENCE, a web-based systematic review platform, a 12-member research team screened studies in three stages: title and abstract screening, full-text review to identify articles relevant to the research questions of our scoping review, and data extraction. Each article was independently reviewed by at least two researchers, within disagreements resolved through discussion. A pre-developed data extraction template was used to collect data from the selected articles, covering participant demographics, examined digital technologies, social support and key findings. Regular online team meetings were held to ensure smooth progression through the different phases of article selection and the final data extraction phase.

## Item 10. Data charting process

To organize and synthesize data effectively to extract information pertinent to our research question the data extraction was conducted entirely within COVIDENCE as it offers robust features for collaboration and comprehensive data handling. The data-charting form for data coding used for this was developed by CMT and calibrated within the team. For each extracted article the data was coded independently by 2 researchers and final consensus was reached during online team meetings.

## Item 11. Data items

Information from the following list of data items and their description will be retrieved from the articles to address the objectives of this scoping review. The name of the reviewer will be written at the top (for internal use only):

| **Item** | **Description** |
| --- | --- |
| Author (APA style) | If 1 author: Author Surname (year). If 2 authors: Author and Author (year). If 3+ authors: First author et al. (year) |
| Year of data collection |  |
| N (number of participants) |  |
| N of participants invited | How many people were interviewed or had questionnaires sent to them in total, no matter if they responded? |
| N in follow-ups | For example, in intervention or randomized control studies: How many participants responded in a follow-up (e.g., after 1 year)? |
| Country |  |
| Type of study | Quantitative, qualitative, mixed |
| Sampling method | How were participants recruited for the research? |
| Data collection method | What type of method was used? e.g. semi-structured interview, in-depth interview, face-to-face questionnaire/survey, telephone survey, online survey, mail survey |
| Data recording method | Pencil-paper, online, audio, video, , secondary data, other |
| Type of population | e.g. employees, employers, general population |
| Type of workers | Occupation/Employment branch |
| Age categories used in analysis/results | If no age categories were analyzed, the age range of the sample |
| Study includes both young and older workers? | Studies with young people may only be included if older workers are also represented and analysed (at least 50 years old) |
| Includes both older adults and older workers? | Studies with older adults who do not work may only be included if older workers are also analysed |
| Gender/sex used in analysis/results | If participants are described for each gender, check each box (”female”, ”male”). If participants are described as whole check ”total f+m” |
| Digital tool | Name / describe the digital tool that was used |
| Digital tools characteristics | Implicit (e.g., remote work, hybrid), Explicit (e.g., apps, digital tools used at work), both (implicit and explicit) |
| Social support | Describe forms and sources of social support |
| Type of social support | Explicit or implicit social support |
| Instruments | Describe instruments/measures/assessments used for the variables digital tools and social support. How were the variables assessed? |
| Main findings | Describe relevant findings for our research goals/questions |
| Theory used | Whether the examination of social support was grounded in a theoretical framework |

## Item 12. Critical appraisal of individual sources of evidence

Although critical appraisal of individual studies is not typically conducted in a scoping review [1], we assessed the quality of the 43 included studies using the Mixed Method Appraisal Tool (MMAT) [2] (see Multimedia Appendix 3). Any discrepancies were resolved through consensus between the reviewers.

## Item 13. Summary measures

Not applicable for scoping reviews.

## Item 14. Synthesis of results

We proposed that findings would be synthesized through descriptive statistics and narrative analysis.

## Item 15. Risk of bias across studies

Not applicable for scoping reviews.

## Item 16. Additional analyses

Not applicable for scoping reviews.

## **Results**

## Item 17. Selection of sources of evidence

The number of sources of evidence screened, assessed for eligibility, and included in the review, with reasons for exclusions at each stage, can be found in the PRISMA flow chart (Figure 1, main text).

## Item 18 Characteristics of sources of evidence

Eligibility was determined based on the PCC framework and additional criteria for inclusion and exclusion is provided in textbox 1 in the main text. A description of the characteristics of the included studies (period of data collection, year of publication, study design, geographic location, sample size of study, type of working population, digital technology and social support) is provided in the main text.

## Item 19 Critical appraisal within sources of evidence

Study quality was assessed using the MMAT [2], which evaluates five study design categories for the sources of evidence: (1) qualitative studies, (2) quantitative randomized controlled trials, (3) quantitative non-randomized studies, (4) quantitative descriptive studies, and (5) mixed methods studies. The MMAT includes a two-part checklist: an initial screening with two questions to confirm the study’s empirical nature (clarity of research questions and feasibility of addressing them), followed by five design-specific criteria guiding the assessment process. Results are provided in Supplemental material_Quality assessment and further described in the main text.

## Item 20 Results of individual sources of evidence

For each included source of evidence, the relevant data that relate to the review questions and objectives are charted in Table 2 and Figure 2.

## Item 21 Synthesis of results

The data is placed in a table and narratively synthesized in the main text, respectively In the subsection “Conceptual map of social support for older workers and impact on health”.

## Item 22 Risk of bias across studies

Not applicable for scoping reviews.

## Item 23 Additional analyses

Not applicable for scoping reviews.

## **Discussion**

## Item 24 Summary of evidence

This scoping review reveals that, despite a growing body of research on digital technologies and work, empirical evidence on how digitalisation shapes social support *specifically for older workers* remains fragmented and limited. Most studies include broad age ranges and rarely provide age-stratified analyses, making it difficult to isolate age-specific mechanisms or outcomes. The evidence base is further constrained by a strong geographical bias towards European and other high-income countries, a dominance of teleworking and generic ICT use as study contexts, and a lack of research in low-wage, manual, or precarious occupations. Social support and digital technology use are operationalised heterogeneously, often through ad hoc or implicit measures, with few studies employing validated multi-dimensional instruments or distinguishing clearly between implicit and explicit, or between emotional, informational, instrumental, and companionship support. Methodologically, the predominance of cross-sectional designs and the concentration of data collection during the COVID-19 period restrict causal inference and generalisability beyond crisis-driven remote work. Finally, evaluated interventions are scarce: there is limited robust evidence on which specific digital tools, organisational practices, or training formats effectively enhance social support and health for older workers over time. Together, these gaps underscore the need for theory-driven, longitudinal, and intervention-focused research that can clarify when and for whom digital technologies function as resources rather than stressors in later-life working careers.

Item 25 Limitations

A potential limitation of this scoping review is that, despite an extensive and systematic search across major academic databases, some relevant studies may have been missed (non-English language publications, very recent publications).

Item 26 Conclusions

Digital technologies enable older workers to receive various forms of social support (e.g., esteem/emotional support, social companionship, informational support, and instrumental support) through platforms that promote communication, such as teleworking, enterprise social media, and health-related apps. These technologies facilitate professional connections and provide mental health benefits, often fostering a sense of inclusion and continuous learning.

However, digitalisation also introduces challenges, especially for those with limited digital skills, potentially leading to social isolation, technostress, and a feeling of inadequacy in fast-paced digital environments. The reviewed studies highlight that, although digital technologies can bolster older workers' productivity and connectivity, the benefits are contingent on adequate training and organisational support.

## Item 27: Funding

The research was partially financed through the COST Action CA21107 “Work inequalities in later life redefined by digitalization” (DIGI-net) that is supported by the European Cooperation in Science and Technology (COST) (<https://www.cost.eu/actions/CA21107/>). The study described is also supported through the project „Research of Excellence on Digital Technologies and Wellbeing CZ.02.01.01/00/22_008/0004583“ which is co-financed by the European Union.

## References

1. Tricco AC, Lillie E, Zarin W, O'Brien KK, Colquhoun H, Levac D, et al. PRISMA Extension for Scoping Reviews (PRISMA-ScR): Checklist and Explanation. Annals of Internal Medicine. 2018;169(7):467-73. <https://doi.org/10.7326/M18-0850>.

2. Hong QN, Fàbregues S, Bartlett G, Boardman F, Cargo M, Dagenais P, et al. The Mixed Methods Appraisal Tool (MMAT) version 2018 for information professionals and researchers. Education for information. 2018;34(4):285-91. <https://doi.org/10.3233/EFI-180221>.
